# Supplementary material for: OT-knn: A neighborhood-aware optimal transport framework for aligning spatial transcriptomics data
Source: PLoS Comput Biol. 2026 Sep 22;22(9):e1014041. doi: 10.1371/journal.pcbi.1014041 (PMC13641709; doi:10.1371/journal.pcbi.1014041)
Supplement: Supplementary file 1 [file pcbi.1014041.s013.pdf]

Point-by-point Responses to Reviewer Comments on PCOMPBIOL-D-26-00380

We sincerely thank Reviewers #1 and 2 for their positive evaluations and constructive comments on our manuscript PCOMPBIOL-D-26-00380 entitled "*OT-knn: a neighborhood-aware optimal transport framework for aligning spatial transcriptomics data*". We have fully addressed all the comments and revised our manuscript accordingly. In the following sections, we provide the point-by-point responses to all comments from each reviewer. Reviewer comments are shown in blue Helvetica font, and our responses are shown in black. All changes in the revised manuscript are shown in teal Helvetica font. All display items, page numbers, and line numbers referenced below correspond to the revised manuscript. Whenever space allows, all the changes introduced in this round of revision are included in this document for the convenience of reviewers.

Reviewer #1 Comments

In this paper, authors proposed a ST alignment method called OT-knn, which integrates local neighborhood information within an optimal transport framework. OT-knn was evaluated using simulated data with known ground-truth alignment and real datasets from multiple ST platforms.

We thank Reviewer #1 for the positive feedback on our manuscript. We are also very grateful for the insightful and constructive comments provided. We have addressed all the comments point by point and revised the manuscript accordingly, as detailed below.

Major comments:

1. The OT-kNN method is based on standard optimal transport (OT), which enforces full mass matching. Consequently, when applied to partially overlapping slices, it may lead to forced correspondences in non-overlapping regions. How is this issue handled in the experimental setup?

Response: We thank the reviewer for raising this important point. We agree that standard balanced optimal transport enforces full mass matching and may therefore introduce forced correspondences when two tissue sections contain substantial non-overlapping regions.

In the experiments reported in this manuscript, the practical impact of this limitation is reduced by the structure of the datasets and the evaluation design. The paired DLPFC and MERFISH sections were selected from comparable anatomical regions and have broadly similar tissue coverage. For the axolotl developmental comparisons, quantitative accuracy was evaluated only for cell types present in both slices; stage-specific cell types without meaningful counterparts were excluded from the evaluation. Similarly, the cross-platform mouse embryo analysis was evaluated using shared major cell-type categories. These choices do not remove the full-mass constraint during optimization, but they focus the reported evaluation on anatomical regions and cell populations for which cross-slice correspondence is meaningful. Under these settings, balanced OT-knn achieved strong and stable empirical performance across the simulated and real datasets examined in the manuscript.

To directly investigate the reviewer's concern, we conducted an additional proof-of-concept analysis using an unbalanced OT extension of OT-knn. The extension retains the same neighborhood-aware feature reconstruction and expression-based cost matrix as OT-knn but replaces the balanced transport step with a KL-relaxed unbalanced OT formulation[1,2]. Rather than strictly enforcing the source and target marginal constraints, the unbalanced formulation penalizes deviations from them. Con-

sequently, spots in non-overlapping or low-confidence regions can carry reduced transport mass instead of being forced to match locations in the other slice. We implemented the analysis and all other preprocessing and OT-knn settings were unchanged.

We evaluated this extension using a partial-overlap simulation constructed from a DLPFC slice pair. We removed the white-matter region from one slice to create a large non-overlapping anatomical domain and removed a corner region containing portions of two cortical layers from the other slice to mimic localized tissue loss. Thus, the simulated pair contained both incomplete anatomical overlap and missing tissue regions.

As shown in Rebuttal Fig. 1, the unbalanced OT extension primarily assigned transport mass within the shared cortical regions and substantially reduced correspondences involving regions without true counterparts. In comparison, methods that produced more complete or less overlap-aware matching exhibited additional errors in the non-overlapping regions. For example, SLAT matched spots from the non-overlapping white-matter region to cortical regions, DeST-OT also mapped some white-matter spots to non-white-matter locations, and PASTE produced more oblique and crossing correspondences between anatomical regions.

This proof-of-concept analysis shows that the neighborhood-aware representation of OT-knn can be combined naturally with unbalanced transport when incomplete overlap is substantial. However, a systematic treatment of partial overlap would require additional investigation of the unbalanced formulation, marginal-relaxation parameters, overlap detection, and validation across a broader range of tissue geometries. Because the datasets analyzed in the present study have largely comparable anatomical coverage and balanced OT-knn performed well empirically, we have retained the balanced formulation as the focus of this manuscript and leave a comprehensive unbalanced or partial-OT extension to future work.

We revised the Discussion to acknowledge this limitation and future direction:

"One limitation of the current framework is its use of balanced OT, which requires the probability mass in each slice to be fully matched to the other. This assumption may lead to forced correspondences when tissue sections contain substantial non-overlapping regions. Extending OT-knn using unbalanced or partial optimal transport, which allows some probability mass to remain unmatched, is therefore an important direction for future work. A systematic treatment of partial overlap will require further investigation of overlap-aware modeling, parameter selection, and validation across diverse tissue geometries."

2.The cost matrix in OT only considers omics-based smooth features and does not incorporate spatial structural constraints. In contrast, PASTE introduces spatial relationships through the Gromov–Wasserstein (GW) distance, while SLAT preserves spatial topology via graph convolution.

In this case, would the current method potentially match spots that are highly similar in feature space but far apart in geometric space, thereby leading to spatially inconsistent or misaligned correspondences?

Response: We thank the reviewer for this insightful question.

While OT-knn does not incorporate raw pairwise spatial distances directly into the optimal transport cost matrix, spatial information is incorporated through the neighborhood reconstruction step prior to optimal transport. Specifically, each spot is represented by a distance-weighted aggregation of the gene expression profiles of its spatial k-nearest neighbors. As a result, the reconstructed feature of

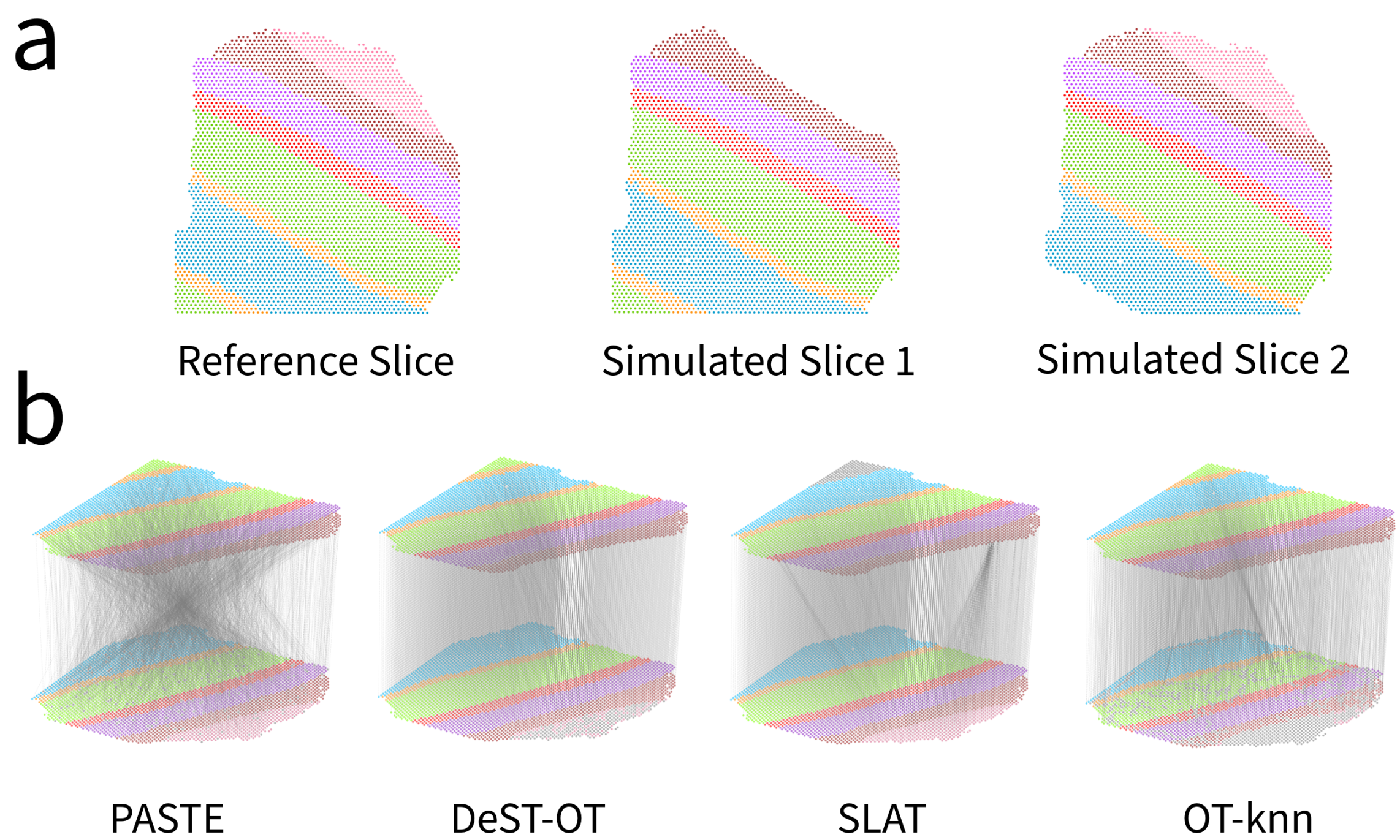

Rebuttal Figure 1: **Alignment for the patial overlap slices simulated from DLPFC data using PASTE, DeST-OT, SLAT, and unbalanced OT-knn. a**. Reference slice (left) and two simulated slices (right). **b**. Visualization of the alignment results for the partially overlapping slices using PASTE, DeST-OT, SLAT, and unbalanced OT-knn. Aligned spots are colored according to their annotated layers (white matter: pink; cortical-layer: other colors) and unaligned spots are shown in gray.

a spot reflects not only its own expression profile but also the local tissue context surrounding that spot.

This neighborhood reconstruction plays an important role in promoting spatially coherent alignments. By aggregating information across neighborhoods of different sizes using distance-weighted averaging, see Equation 1 in the manuscript, the reconstructed features capture tissue context at multiple spatial scales, ranging from immediate neighbors to the broader local microenvironment. Consequently, spots that are spatially distant but happen to have similar individual expression profiles are less likely to have similar reconstructed neighborhood profiles. The OT cost matrix is therefore computed on features that already encode spatial organization, encouraging spatially coherent alignments without explicitly imposing coordinate-based constraints in the transport objective.

This idea is conceptually related to SLAT, as both leverage local neighborhood structure to improve spatial consistency. However, SLAT learns spatially informed embeddings through graph convolution, whereas OT-knn uses an explicit distance-weighted neighborhood aggregation step before optimal transport, producing interpretable microenvironment-aware representations.

A potential advantage of this design is that it relies on relative neighborhood structure rather than absolute coordinates. Direct incorporation of coordinates can make alignment sensitive to geometric differences between slices, such as local deformation, rotation, translation, or nonlinear distortion. In contrast, neighborhood-based representations tend to be more stable under such perturbations

because local tissue context is often preserved even when global geometry changes.

This behavior is supported by our empirical results. Across the DLPFC, MERFISH, and axolotl datasets, OT-knn achieves strong mapping-probability and best-matching accuracy, with most alignment errors concentrated near tissue-domain boundaries rather than between anatomically distant regions. In addition, in the new coordinate-perturbation simulation included in Rebuttal Figure 2 for answering Reviewer 1, Question 3, OT-knn remains more robust to coordinate rotation and deformation than competing approaches. These results suggest that neighborhood-aware feature reconstruction effectively preserves spatial consistency while maintaining robustness to geometric variation across slices.

3.In OT-kNN, structural noise is simulated using a mild nonlinear transformation of the form ($x^{1.02}$,$y^{1.01}$). However, this transformation is separable along the two coordinate axes and does not introduce any local deformation, shear, or topological changes. Moreover, since the exponents are only slightly greater than 1, the transformation is numerically close to a linear mapping, resulting in a relatively small overall perturbation. Such a design is insufficient to capture the non-rigid deformations commonly observed in real biological tissues, such as local twisting, folding, or complex misalignment between slices. Under this limited nonlinear setting, the robustness of the method to structural noise may be overestimated. Furthermore, the experimental results presented in the manuscript do not provide convincing evidence that OT-kNN outperforms methods such as SLAT or DeST-OT under structural noise. In particular, experiments involving more complex structural inconsistencies are lacking.

Response: This question is closely related to question #4, so we respond to both questions together in question #4.

4. The experiments presented in the manuscript only consider non-rigid transformations, and it is unclear whether the authors have evaluated the method under rigid transformations, such as rotation and translation between slices.

Response: We thank the reviewer for this important suggestion. We agree that the nonlinear transformation used in the original manuscript represents only a mild structural perturbation and does not capture more complex geometric inconsistencies that can occur between tissue sections. Our original simulations focused on nonlinear distortions because rigid transformations such as rotation and translation are generally less challenging for OT-based alignment methods. Nevertheless, we agree that explicitly evaluating both rigid and non-rigid perturbations provides a more complete assessment of alignment robustness.

To address these concerns, we added a substantially more challenging simulation framework (Rebuttal Figure 2a). Specifically, we introduced both global and local structural perturbations. Global perturbations consisted of a 45° rotation together with translations corresponding to 20% and 15% of the tissue width and height, respectively. To model local non-rigid deformation, we divided the tissue into a 5×5 grid and applied independent random rotations (up to 6°) and translations (up to 2% of tissue size) to each patch. We further introduced gene-expression perturbations following the simulation design in the manuscript and incorporated spot dropout to mimic missing or low-quality capture spots.

Under this more realistic setting, OT-knn achieved the highest best-matching accuracy among all methods evaluated (PASTE, DeST-OT, and SLAT; Rebuttal Figure 2b). Visualization of one-to-one matches (Rebuttal Figure 2c) further shows that OT-knn produces fewer mismatched and unmatched spots than competing methods. These new experiments demonstrate that OT-knn remains robust

under both rigid transformations (rotation and translation) and more complex local structural deformations. The results have been added to the revised manuscript in the section "Simulation-based evaluation under spatial and transcriptional perturbations".

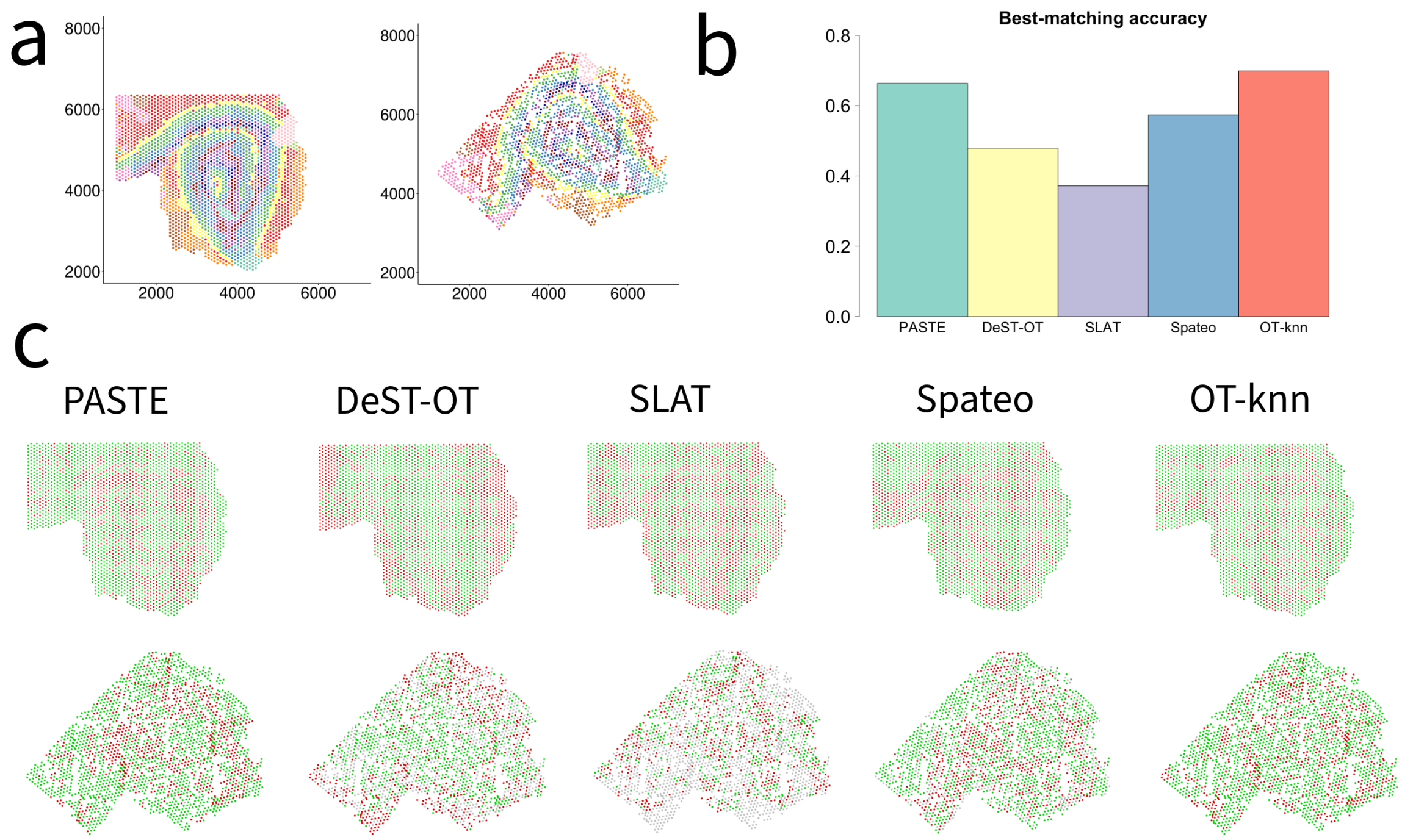

Rebuttal Figure 2: **Performance comparison under controlled perturbations.** **a**. Reference slice (left) and perturbed slice (right), colored by clusters obtained using Seurat. **b**. Best-matching accuracy for PASTE, DeST-OT, SLAT, Spateo and OT-knn. **c**. One-to-one matching results. Correctly aligned spots are shown in green, misaligned spots in red, and unaligned spots in gray. The top row shows the reference slice and the bottom row shows the perturbed slice.

5. All experiments in the manuscript are conducted on datasets generated from the same platform. It remains unclear whether the proposed method can effectively align slices obtained from different sequencing platforms.

Response: We thank the reviewer for this suggestion. We agree that, while the original manuscript evaluated datasets generated by multiple spatial transcriptomics technologies (Visium, MERFISH, and Stereo-seq), it did not directly assess cross-platform alignment between slices generated by different technologies.

To address this limitation, we performed an additional cross-platform alignment experiment using mouse embryo datasets generated by Stereo-seq and seqFISH (Rebuttal Figure 3). This setting is particularly challenging because the two platforms differ substantially in gene coverage, spatial resolution, and cell-type annotation granularity. To enable evaluation, we mapped the original annotations to seven broad cell-type categories shared across both datasets.

Despite these substantial platform differences, OT-knn achieved the highest best-matching accuracy and mapping probability accuracy among all methods evaluated (PASTE, DeST-OT, Spateo, and SLAT), with an accuracy of 0.42 and 0.34, respectively (Rebuttal Figure 3b). Although this accuracy is lower than those observed in our same-platform analyses, this reduction is expected given the

additional challenges of cross-platform alignment. These results demonstrate that OT-knn remains effective when aligning slices generated by different spatial transcriptomics technologies.

This new analysis has been added to section "Cross-platform alignment between Stereo-seq and seqFISH mouse embryo datasets" in the revised manuscript.

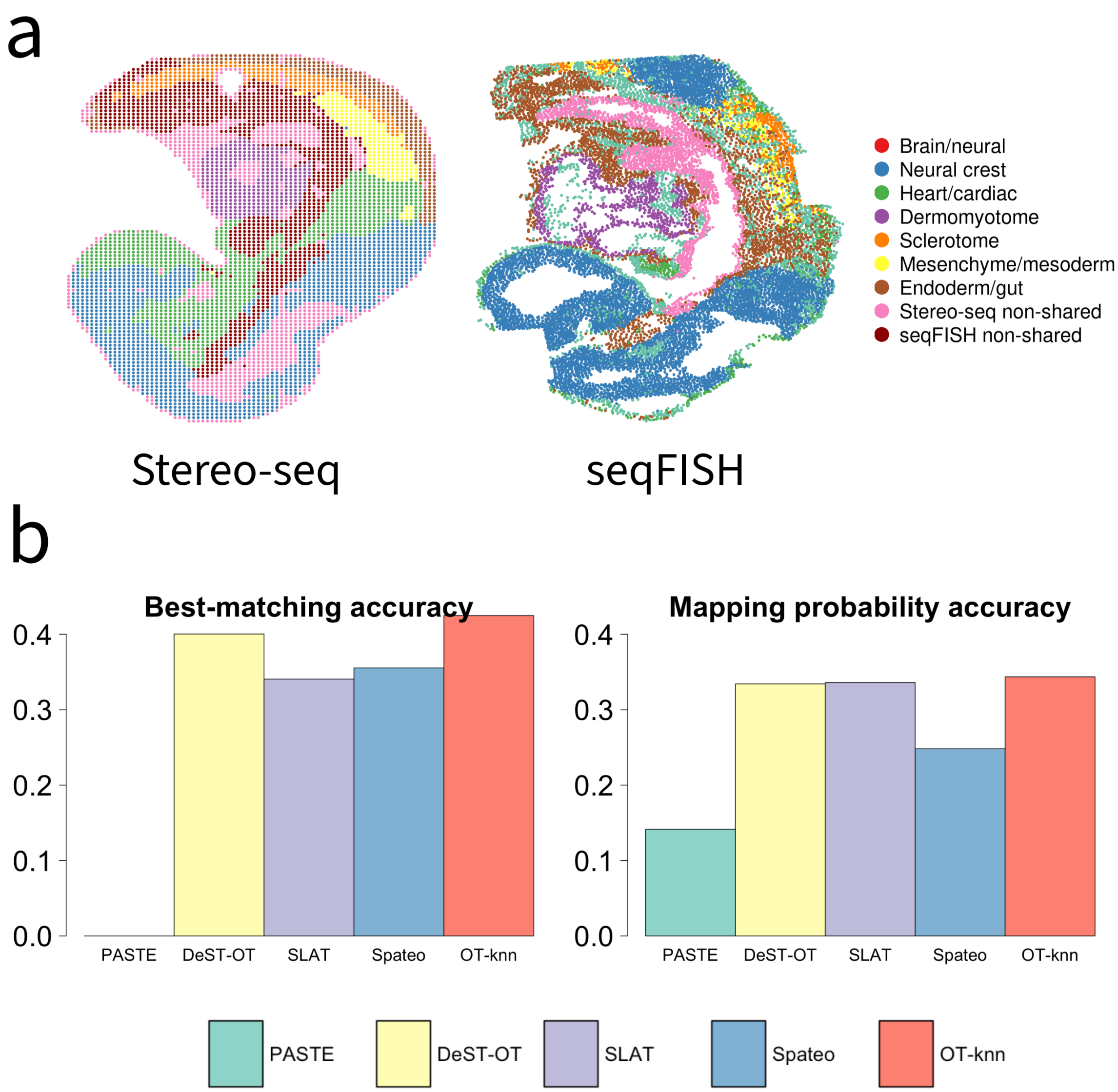

Rebuttal Figure 3: **Performance comparison for mouse embryo datasets by Stereo-seq and seqFISH. a**. Visualization of E9.5 (Stereo-seq) and E8.75 (seqFISH) mouse embryo datasets, colored by cell types. **b**. Best-matching accuracy and mapping probability accuracy for PASTE, DeST-OT, SLAT, Spateo, and OT-knn.

6. In the benchmarking experiments, the reproduction of SLAT obtains matching by applying optimal transport (OT) on the learned embeddings. However, this deviates from the original SLAT methodology, which determines correspondences directly based on embedding similarity. Why is OT applied as an additional step instead of using the original matching strategy proposed in SLAT? Notably, SLAT itself is designed to perform alignment through its embedding and matching

mechanism, and introducing OT may alter its intended behavior and potentially affect the fairness of the comparison.

Response: We thank the reviewer for raising this important point. We would like to clarify that we used SLAT's original matching output directly for all best-matching evaluations and visualizations. Specifically, the best-matching accuracy comparisons and matching visualizations in Figs. 2–5 were based on SLAT's native one-to-one correspondences and did not involve an additional OT step.

The OT step was used only for computing mapping-probability accuracy. Unlike PASTE, DeST-OT, and OT-knn, which naturally produce a probabilistic mapping matrix, SLAT outputs spot embeddings and best-matching pairs but does not provide probabilistic alignment scores. Because mapping-probability accuracy is defined on a probability matrix, we constructed an OT-based probability matrix from SLAT's learned embeddings solely for this evaluation metric. This procedure was not used for generating SLAT's matches, visualizations, or best-matching accuracy results.

We have clarified this point in the Benchmarking Methods section of the revised manuscript as follows: (revised text is in teal, and replaced text is crossed out)

"PASTE and DeST-OT produce a probabilistic mapping matrix $T = [T_{ij}]$ that quantifies alignment probabilities between spots across slices. For these methods, one-to-one correspondences were obtained by assigning each spot to the counterpart with the highest mapping probability. ~~SLAT outputs spot embeddings and best-matching pairs but does not provide a probabilistic mapping. To enable evaluation using mapping probability accuracy, we constructed a probability matrix for SLAT by computing an optimal transport plan between the two embedding spaces, minimizing the Wasserstein distance as in Equation 2. This procedure yields a probabilistic alignment that is directly comparable to the outputs of OT-based methods.~~ SLAT directly outputs spot embeddings and one-to-one matching pairs but does not provide a probabilistic mapping matrix. Therefore, all best-matching accuracy evaluations and matching visualizations for SLAT were based on its original matching output. To enable evaluation using mapping-probability accuracy, which requires a probabilistic mapping matrix, we additionally constructed a probability matrix from the SLAT embeddings by computing an optimal transport plan between the two embedding spaces, minimizing the Wasserstein distance as in Equation 2. This OT-derived probability matrix was used only for calculating mapping-probability accuracy and did not affect SLAT's matching results and visualizations. "

7. In the experiments on the DLPFC dataset, the results reported for PASTE are inconsistent with those presented in the original paper. This discrepancy raises concerns about the correctness of the implementation or the experimental setup used in this study.

Response: We thank the reviewer for raising this point and for the opportunity to clarify the discrepancy between our reported PASTE results and those presented in the original PASTE study.

The discrepancy arises from the use of different initialization strategies rather than from any issue with our implementation or experimental setup. Specifically, the DLPFC results reported in the original PASTE paper were generated using a geometry-based initialization included in the authors' DLPFC reproducibility code but not in the standard PASTE workflow described in the tutorial. This initialization constructs an initial transport plan from the spatial coordinates before performing alignment. (Specifically, this step first normalizes and centers the spatial coordinates of the two slices, computes a Euclidean distance matrix, and solves an OT problem based only on spatial distance to generate the initial transport plan "`G_init`".) To our knowledge, this initialization was used in the DLPFC reproducibility code but does not appear in the analyses of the other datasets reported in the PASTE paper.

To verify this, we reran PASTE using the authors' DLPFC-specific reproducibility code and successfully reproduced the results reported in the original publication. We also reproduced the published PASTE results for other datasets using the standard workflow (e.g., the SCC (human squamous cell carcinoma) dataset). These findings demonstrate that the discrepancy is attributable to the choice of initialization rather than to an implementation error or an incorrect experimental setup.

We further observed that the geometry-based initialization had the largest effect on the A–B and C–D pairs in Samples I and II, while its effect on the B–C pairs was much smaller. Because A–B and C–D correspond to adjacent slices (10μm apart), whereas B–C pairs are separated by a substantially larger distance (300 μm apart), this observation suggests that the impact of the initialization may depend on the degree of similarity between the slices being aligned. Because this initialization is dataset-specific and not part of the standard PASTE workflow, we chose not to use it in order to maintain a consistent and method-agnostic comparison across all methods.

We have clarified this point in the revised manuscript and now explicitly note that the original DLPFC results can be reproduced when the DLPFC-specific initialization is used. We added the following clarification in the section "Benchmarking methods":

"For all datasets, we ran PASTE using its standard workflow and default initialization of the transport plan. This differs from the publicly available implementation used for the PASTE analysis of the DLPFC dataset, which employs a dataset-specific, geometry-informed initialization constructed from the spatial coordinates of the paired slices. This tailored initialization can be advantageous when the slices have similar global geometry, orientation, and anatomical coverage, but it may be less broadly applicable when these properties differ. Moreover, the other benchmarked methods do not use comparable dataset-specific initialization. We therefore used the standard PASTE workflow to maintain a consistent and non-tailored benchmarking procedure across methods and datasets."

8. The results in Fig. 3e show that increasing the number of neighboring nodes consistently improves model performance. However, in graph-based models, incorporating too many neighbors typically leads to over-smoothing, where node representations become indistinguishable and performance degrades. It is therefore unclear whether over-smoothing occurs in this setting, and how its impact on model performance is evaluated. The current results may not fully capture this effect.

Response: We thank the reviewer for this insightful comment. Classical over-smoothing is typically associated with graph neural networks that repeatedly propagate information across a graph, causing node representations to become increasingly similar. OT-knn differs from this setting because it performs a single distance-weighted neighborhood aggregation rather than iterative graph propagation.

To directly assess whether large neighborhood sizes lead to over-smoothing, we expanded our sensitivity analysis from the original range of (k = 50–150) to (k = 5–360) (Rebuttal Figure 4). We observed that alignment performance improved as k increased from very small values ($k < 40$), but plateaued once ($k > 60$). Importantly, we did not observe the performance degradation that would be expected under severe over-smoothing.

One likely reason is that OT-knn uses distance-weighted aggregation, so nearby neighbors contribute much more strongly than distant neighbors even when k is large. As a result, the reconstructed representation remains dominated by the local microenvironment rather than converging toward a global average.

These results indicate that OT-knn is relatively insensitive to the choice of k over a broad range and that substantial over-smoothing was not observed in the datasets examined. We have updated the Fig. 3e, now presented as Fig. 3f, and added this expanded sensitivity analysis to the Supplementary Materials.

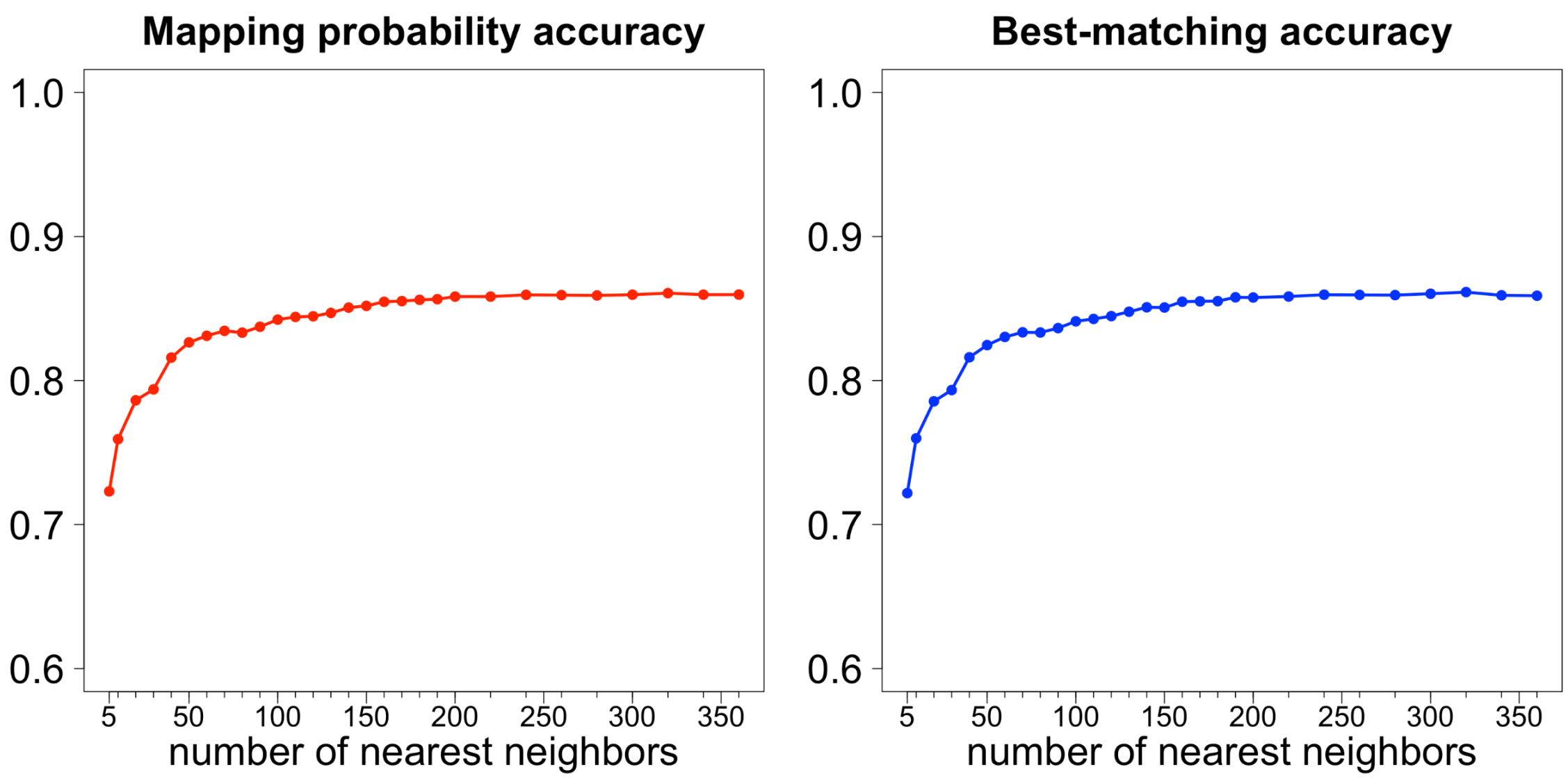

Rebuttal Figure 4: **Mapping probability accuracy and best-matching accuracy across different numbers of nearest neighbors for the middle pair of DLPFC Sample II.**

9. It would be beneficial to include additional methods in the benchmarking experiments to provide a more comprehensive and convincing evaluation.

Response: We thank the reviewer for this helpful suggestion. We agree that including additional methods strengthens the evaluation.

In the original manuscript, we compared OT-knn with PASTE, DeST-OT, and SLAT because these methods represent several major classes of spatial transcriptomics alignment approaches, including optimal transport–based methods, developmental alignment methods, and graph-based methods. To further expand the benchmark, we reviewed a recent published benchmark paper that provides comprehensive evaluations of spatial transcriptomics alignment methods[3]. This benchmark identified Spateo[4] as one of the strongest and most robust methods across diverse datasets and perturbation settings. Spateo jointly models molecular profiles and spatial coordinates and has demonstrated competitive performance in spatial transcriptomics alignment and three-dimensional reconstruction tasks. We therefore included Spateo as an additional state-of-the-art baseline in our revised experiments.

We therefore added Spateo as an additional baseline and evaluated it on the simulation, DLPFC, MERFISH, and Stereo-seq datasets. The corresponding results have been added to the revised manuscript and Supplementary Materials. Across these datasets, OT-knn consistently achieved comparable or better performance than Spateo.

Overall, the inclusion of Spateo broadens the benchmark with a recently developed state-of-the-art method and further supports the robustness of OT-knn across diverse alignment settings.

Reviewer #2 Comments

In this manuscript, the authors developed OT-knn, a neighborhood-aware optimal transport framework designed to align spatial transcriptomics (ST) data across different tissue slices. By reconstructing the gene expression profile of each spot based on its spatial k-nearest neighbors, the method aims to improve robustness to noise, spatial deformation, and biological heterogeneity. The authors systematically evaluated OT-knn on simulated and real datasets from 10x Genomics Visium, MERFISH, and Stereo-seq platforms, benchmarking it against methods such as PASTE, DeST-OT, and SLAT.

We thank Reviewer #2 for the positive feedback on our manuscript. We are also very grateful for the insightful and constructive comments provided. We have addressed all the comments in full and revised the manuscript accordingly, as detailed below.

Major comments:

1. Methods such as PASTE can infer OT-based pairwise correspondences across multiple slices to reconstruct 3D tissue. The authors should demonstrate whether OT-knn's neighborhood-aware alignment leads to more accurate or more spatially consistent 3D stacking compared to existing approaches, or at least discuss its potential and limitations in this regard.

Response: We thank the reviewer for this insightful suggestion. Although OT-knn is designed for pairwise alignment, the probabilistic transport matrices produced by OT-knn can naturally be used for sequential multi-slice stacking, similar to existing OT-based approaches such as PASTE.

To illustrate this capability, we used OT-knn-derived pairwise correspondences to sequentially align four adjacent slices from DLPFC Sample III and reconstruct a three-dimensional tissue structure. As shown in Rebuttal Figure 5, the reconstructed volume preserves the cortical-layer organization and exhibits spatially coherent alignments across slices. These results demonstrate the potential of OT-knn for multi-slice reconstruction. We have added this analysis as Fig. 3g and a corresponding discussion to the revised manuscript in section "Human DLPFC data (10X Genomics Visium)", subsection "Multi-slice reconstruction and domain identification".

2. Beyond pairwise alignment, existing methods such as PASTE and SLAT have demonstrated the ability to integrate multiple slices for simultaneous spatial domain identification across slices. The authors should evaluate whether OT-knn's multi-slice alignment yields more consistent spatial domain, and evaluate it using ARI and NMI. Furthermore, the authors should benchmark OT-knn against dedicated multi-slice integration tools like STAligner (https://www.nature.com/articles/s43588-023-00528-w).

Response: We thank the reviewer for this helpful suggestion. To address this point, we extended OT-knn from pairwise alignment to a multi-slice domain-consistency analysis using the four slices of DLPFC Sample III. Pairwise alignments between adjacent slices (A–B, B–C, and C–D) were first used to identify corresponding spots and construct matched spot chains spanning all four slices. Gene expression profiles of spots belonging to the same chain were jointly embedded by PCA into a shared lower dimensional space, and the chain-level embeddings were clustered to obtain domain labels that were subsequently assigned back to the corresponding spots. (Rebuttal Figure 6).

Following the reviewer's suggestion, we evaluated domain consistency using adjusted Rand index (ARI) and normalized mutual information (NMI). OT-knn achieved an average ARI of 0.577 and an average NMI of 0.672. This is comparable with the dedicated multi-slice integration method STAligner[5], which reports an average ARI of 0.614 for STAligner and 0.364 for PASTE. These results

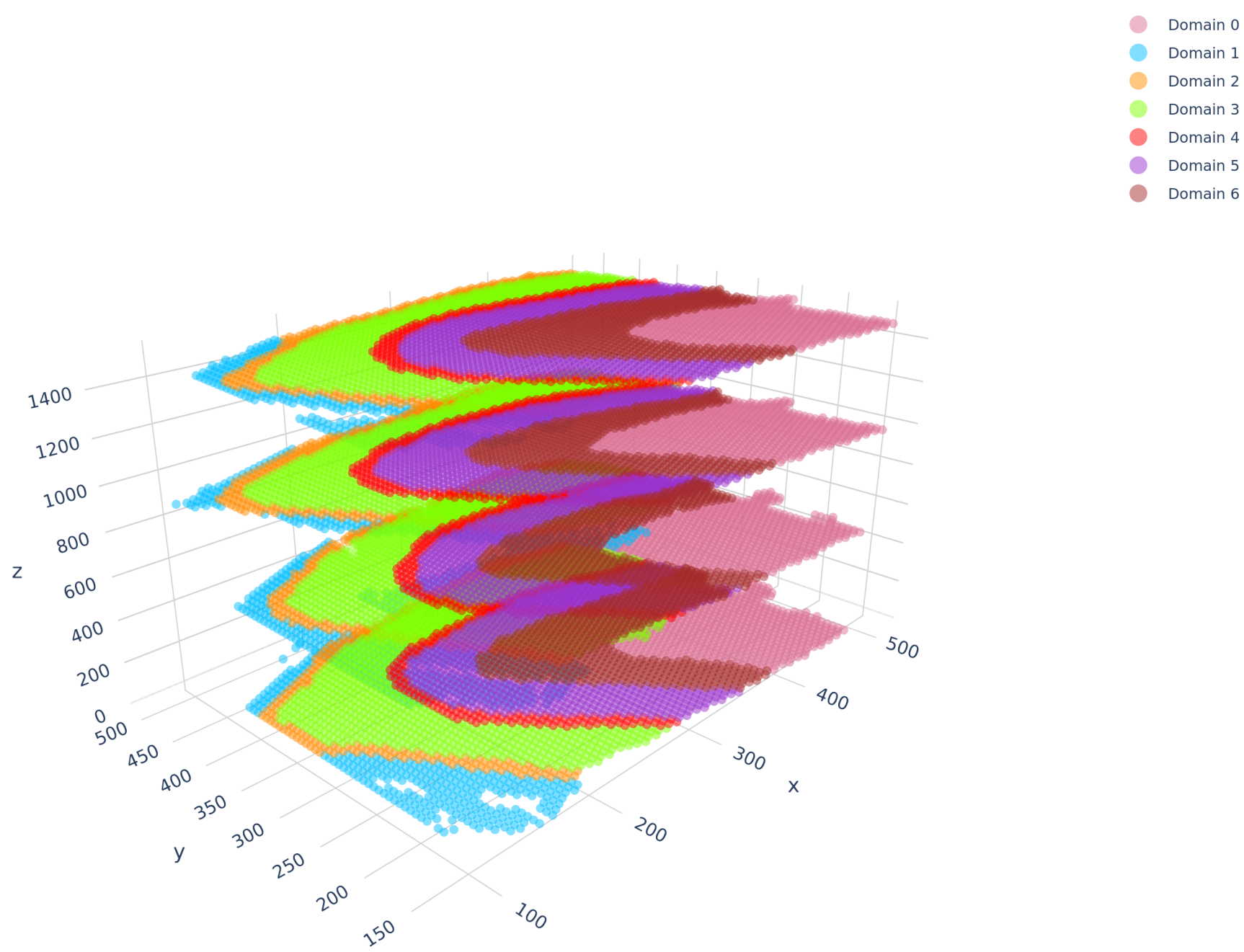

Rebuttal Figure 5: **Stacked 3D alignment of the four tissue slices of DLPFC sample III after alignment with OT-knn.**

indicate that OT-knn can support downstream multi-slice integration and yields spatial domain assignments comparable to those obtained by a dedicated integration method.

We have added this analysis and the corresponding discussion to the revised manuscript and Supplementary Materials.

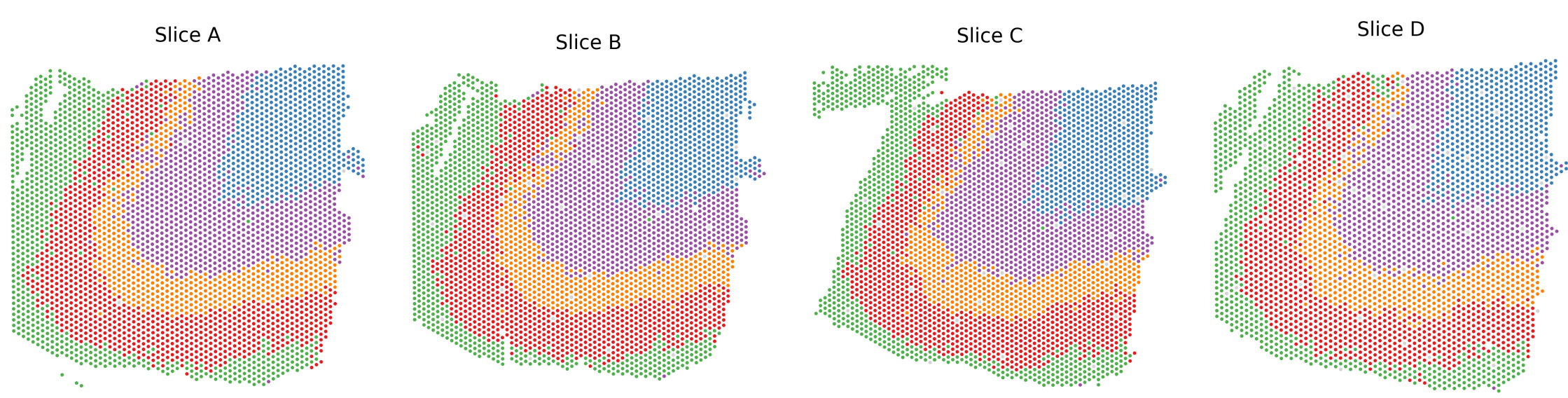

Rebuttal Figure 6: **Domain-identification results for the four slices of DLPFC Sample III.**

3. The simulation framework may not fully capture realistic spatial and transcriptional perturbations. The authors generate the second slice from a single human intestine slice using global spatial distortion and additive expression noise. Real tissue slices often exhibit local, non-linear deformations and complex batch effects. I suggest the authors either validate on additional simulated scenarios (e.g., patch-wise distortion, dropout noise) or explicitly discuss this limitation.

Response:

We thank the reviewer for this helpful suggestion. We agree that the original simulations do not

capture the full complexity of spatial and transcriptional variation observed in real tissue sections. The primary purpose of these controlled simulations is not only to compare alignment accuracy under known ground truth, but also to isolate the effects of different perturbation types and reveal method-specific sensitivities. Specifically, the original scenarios separately introduced spatial deformation, gene expression variation, and their combination, allowing us to examine how each source of variation affects the behavior of the evaluated methods. Robustness to more complex biological heterogeneity was additionally assessed using the real datasets.

To address the reviewer's concern, we expanded the simulation framework with a fourth, more challenging scenario that combines several sources of variation commonly encountered across tissue sections (Rebuttal Fig. 2a). Starting from a human intestine slice, we first applied a global rigid transformation consisting of a 45° rotation and translations corresponding to 20% and 15% of the tissue width and height, respectively, to mimic overall slice misalignment. We then divided the tissue into a 5×5 grid and applied independent local rotations of up to 6° and translations of up to 2% of the tissue size to each patch, thereby introducing patchwise non-rigid deformation. To model transcriptional variation and batch-related noise, gene counts at each spot were multiplied by a random factor between 0.7 and 1.3, followed by the addition of random noise ranging from 0 to 10 counts. Finally, low-count spots were preferentially removed to mimic missing or low-quality capture locations and incomplete local overlap.

The first three simulations therefore provide controlled, diagnostic settings for understanding the sensitivity of each method to individual perturbations, whereas the new fourth scenario evaluates robustness when multiple spatial and transcriptional perturbations occur simultaneously. Under this composite setting, OT-knn achieved the highest best-matching accuracy among PASTE, DeST-OT, SLAT, Spateo, and OT-knn (Rebuttal Fig. 2b). Visualization of the one-to-one correspondences further showed that OT-knn produced fewer mismatched and unmatched spots than the competing methods (Rebuttal Fig. 2c). These findings support the robustness of the neighborhood-aware representation under simultaneous global misalignment, local non-rigid deformation, transcriptional variation, and spot dropout.

We have added this new simulation to the revised Methods and Results sections. The revised manuscript now distinguishes the first three controlled scenarios, which isolate method-specific sensitivities, from the fourth composite scenario, which provides a more stringent and realistic robustness assessment.

4. Optimal transport yields a probabilistic mapping between tissue slices, which is a core strength of OT-knn. However, the paper primarily visualizes the final discrete one-to-one matches (green/red plots). To help readers better understand the structure of the probabilistic transport plan, I suggest the authors add visualizations analogous to SLAT's Fig. 3a and Fig. 4a for the real dataset.

Response: We thank the reviewer for this helpful suggestion. We agree that the probabilistic transport plan is a key output of OT-knn and that visualizing it directly provides additional insight beyond discrete one-to-one matches. Following the reviewer's suggestion, we added transport-plan visualizations for the DLPFC dataset using a style analogous to that used in SLAT (Rebuttal Figure 7). These visualizations illustrate that OT-knn produces spatially coherent correspondences between anatomically similar regions across slices.

We note that the transport-plan visualization and our original two-dimensional one-to-one matching plots provide complementary perspectives. While transport-plan visualizations highlight the overall structure of the probabilistic correspondences, the two-dimensional match plots more clearly reveal mismatched and unmatched spots, particularly when the paired slices differ substantially. For

this reason, we retain the original visualizations and supplement them with transport-plan visualizations. Similar visualizations for the MERFISH dataset have been added to the Supplementary Materials (S5 Fig).

We have incorporated these new visualizations into the revised manuscript (Fig. 3e) and Supplementary Materials.

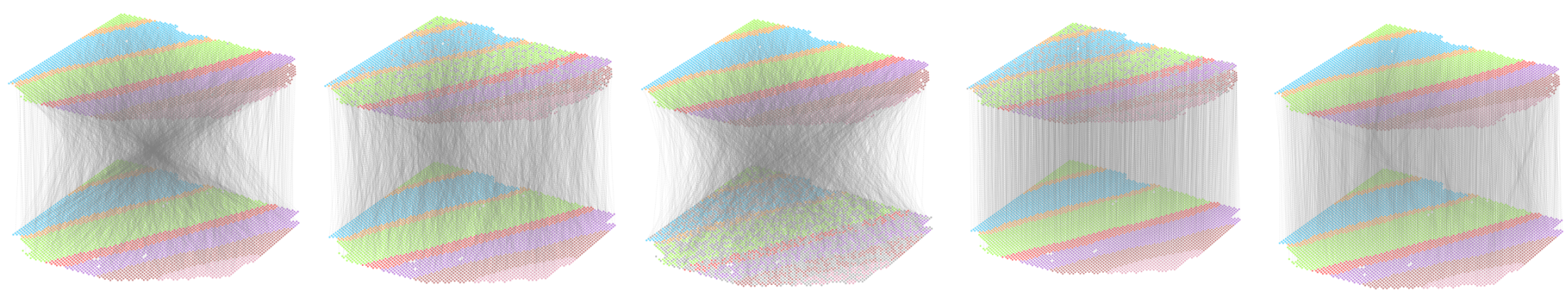

Rebuttal Figure 7: **Visualization of the alignment for the first slice pair of Sample I in the DLPFC dataset.** Spots are colored according to their annotated cortical layers, and unaligned spots are shown in gray. Connection lines represent the best-matching spots across the two slices.

5. Scalability of OT-knn to ultra-high-resolution technologies. Emerging platforms like Visium HD and Xenium generate data at subcellular or near-single-cell resolution, resulting in significantly more data points. The computational cost of building a kNN graph and solving an OT problem for hundreds of thousands of cells is nontrivial. The authors should discuss the expected runtime and memory scaling of OT-knn.

Response: We thank the reviewer for raising this important point. We agree that scalability is an important consideration for emerging ultra-high-resolution platforms such as Visium HD and Xenium.

We expanded the computational-efficiency comparison to include Spateo in addition to PASTE, DeST-OT, SLAT, and OT-knn (Supplementary Table S1). Across the datasets examined, which contained up to approximately 17,500 spots per slice pair, OT-knn completed all analyses on CPUs and had runtimes broadly comparable to DeST-OT, while being faster for several of the larger MERFISH comparisons. Spateo generally required less CPU time, whereas SLAT was faster but relied on GPU acceleration. These results indicate that the current OT-knn implementation is computationally practical for the dataset sizes evaluated in this study.

The computational cost of OT-knn consists of two main components. Construction and storage of the spatial k-nearest-neighbor graph are relatively efficient because the graph is sparse and requires $O(nk)$ memory for a slice with n spots. This step can also be accelerated using approximate nearest-neighbor search[6].

The main computational cost arises from the optimal transport step. For two slices containing n and m spots, the dense pairwise cost matrix and transport plan require $O(nm)$ memory. This computational requirement is shared by standard OT-based alignment methods and is not specific to OT-knn. Recent advances in optimal transport provide several strategies for improving scalability. Entropic regularization with Sinkhorn optimization[7] can accelerate transport computation, whereas

low-rank OT approaches such as FRLC[8] can reduce memory usage through factorized representations of the transport plan. These approaches could be incorporated into OT-knn in future work to support increasingly large and high-resolution spatial transcriptomics datasets.

We have added this discussion to the revised manuscript.

"Scalability will become increasingly important as technologies such as Visium HD and Xenium generate datasets at near-single-cell or subcellular resolution. The sparse spatial k-nearest-neighbor graph in OT-knn can be constructed efficiently using approximate nearest-neighbor search[6]. As in other OT-based alignment methods, the main computational cost arises from the dense pairwise cost and transport matrices, which require $O(nm)$ memory for slices containing n and m spots. Entropic regularization with Sinkhorn optimization[7] and low-rank OT approaches such as FRLC[8] offer practical directions for improving the computational and memory efficiency of this step."

References

[1] Lenaic Chizat, Gabriel Peyré, Bernhard Schmitzer, and François-Xavier Vialard. Scaling algorithms for unbalanced transport problems. *arXiv preprint arXiv:1607.05816*, 2016.

[2] Charlie Frogner, Chiyuan Zhang, Hossein Mobahi, Mauricio Araya, and Tomaso Poggio. Learning with a wasserstein loss. *Advances in neural information processing systems*, 28, 2015.

[3] Yunzhi Yan, Tianyi Gu, Chengcheng Sun, Yinghao Zhang, Yan Cui, Senlin Lin, Qi Zou, Yixuan Du, Chuangyi Han, Kairan Kang, et al. Benchmarking alignment methods for spatial transcriptomics data. *Nature Computational Science*, pages 1–18, 2026.

[4] Xiaojie Qiu, Daniel Y Zhu, Yifan Lu, Jiajun Yao, Zehua Jing, Kyung Hoi Min, Mengnan Cheng, Hailin Pan, Lulu Zuo, Samuel King, et al. Spatiotemporal modeling of molecular holograms. *Cell*, 187(26):7351–7373, 2024.

[5] Xiang Zhou, Kangning Dong, and Shihua Zhang. Integrating spatial transcriptomics data across different conditions, technologies and developmental stages. *Nature Computational Science*, 3(10):894–906, 2023.

[6] Yu A Malkov and Dmitry A Yashunin. Efficient and robust approximate nearest neighbor search using hierarchical navigable small world graphs. *IEEE transactions on pattern analysis and machine intelligence*, 42(4):824–836, 2018.

[7] Marco Cuturi. Sinkhorn distances: Lightspeed computation of optimal transport. *Advances in neural information processing systems*, 26, 2013.

[8] Peter Halmos, Xinhao Liu, Julian Gold, and Benjamin J Raphael. Low-rank optimal transport through factor relaxation with latent coupling. *Advances in Neural Information Processing Systems*, 37:114374–114433, 2024.
