# Supplementary material for: OT-knn: A neighborhood-aware optimal transport framework for aligning spatial transcriptomics data
Source: PLoS Comput Biol. 2026 Sep 22;22(9):e1014041. doi: 10.1371/journal.pcbi.1014041 (PMC13641709; doi:10.1371/journal.pcbi.1014041)
Supplement: Supplementary file 2 [file pcbi.1014041.s011.docx]

**Multi-slice analysis of DLPFC Sample III**

We applied the multi-slice procedure described in the Methods to the four anatomically ordered slices (A-D) of DLPFC Sample III. OT-knn was first applied to the three consecutive slice pairs, A-B, B-C, and C-D, to obtain pairwise probabilistic mappings and best-matching spot correspondences.

For domain identification, best-matching correspondences from the three consecutive alignments were linked transitively to construct spot chains across the four slices. Only chains containing one spot from every slice were retained. Following the preprocessing described in the Methods, the expression matrices from the four slices were concatenated and projected into a common latent space using principal component analysis (PCA). The top 30 principal components were retained. For each spot chain, the PCA embeddings of its constituent spots were averaged to obtain a chain-level representation.

We examined clustering solutions with K=5-7. The K=5 solution produced more spatially coherent domains that were consistently represented across slices, whereas the K=6 and K=7 solutions included some clusters that spanned multiple annotated cortical layers. We therefore used K=5 for the subsequent visualization and quantitative evaluation. This empirically selected value was not intended to impose a one-to-one correspondence between the inferred expression domains and the six annotated cortical layers plus white matter, because transcriptionally similar anatomical regions may be grouped into the same expression-derived domain.

The inferred domains were spatially coherent across the four slices and showed substantial agreement with the annotated cortical layers (S1 Fig). Quantitative comparison with the annotations yielded an adjusted Rand index (ARI) of 0.577 and a normalized mutual information (NMI) of 0.672. For context, Zhou et al. [1] reported an ARI of 0.614 for STAligner, which was developed specifically for joint multi-slice integration, compared with 0.364 for PASTE on the same DLPFC sample. Because the analyses may differ in preprocessing, clustering, and parameter selection, these values are provided as contextual information rather than as a direct head-to-head comparison. Overall, these results indicate that correspondences obtained from consecutive OT-knn alignments preserve sufficient cross-slice structure to support domain identification across multiple tissue sections.

Reference

1. Zhou X, Dong K, Zhang S. Integrating spatial transcriptomics data across different conditions, technologies and developmental stages. Nature Computational Science. 2023;3(10):894-906.
