# Supplementary material for: OT-knn: A neighborhood-aware optimal transport framework for aligning spatial transcriptomics data
Source: PLoS Comput Biol. 2026 Sep 22;22(9):e1014041. doi: 10.1371/journal.pcbi.1014041 (PMC13641709; doi:10.1371/journal.pcbi.1014041)
Supplement: Supplementary file 3 [file pcbi.1014041.s010.doc]

**Sensitivity to neighborhood size**

We evaluated the sensitivity of OT-knn to the neighborhood size by varying *k* from 5 to 360 using the B-C slice pair from Sample II of the DLPFC dataset. Both alignment metrics improved as *k* increased from very small values and stabilized at approximately *k*=60, remaining similar across a broad range of larger neighborhood sizes.

This stability is supported by the distance-weighted aggregation used in OT-knn, which assigns greater influence to nearby spots than to more distant neighbors. Consequently, even when *k* is large, the reconstructed expression profile remains primarily determined by the local tissue context rather than approaching a global average. Overall, the performance is largely insensitive to further increases once *k* is large enough, with no substantial performance decline attributable to over-smoothing over the range examined.
