# Supplementary material for: OT-knn: A neighborhood-aware optimal transport framework for aligning spatial transcriptomics data
Source: PLoS Comput Biol. 2026 Sep 22;22(9):e1014041. doi: 10.1371/journal.pcbi.1014041 (PMC13641709; doi:10.1371/journal.pcbi.1014041)
Supplement: Peer review history

# Peer review: Decision Letter 0

**DOI:** 10.1371/journal.pcbi.1014041.r001

**Authors:** Can Yang, Lihua Zhang

30 Apr 2026

OT-knn: a neighborhood-aware optimal transport framework for aligning spatial transcriptomics data

PLOS Computational Biology

Dear Dr. Li,

Thank you for submitting your manuscript to PLOS Computational Biology. After careful consideration, we feel that it has merit but does not fully meet PLOS Computational Biology's publication criteria as it currently stands. Therefore, we invite you to submit a revised version of the manuscript that addresses the points raised during the review process.

Please submit your revised manuscript by June 29, 2026. If you will need more time than this to complete your revisions, please reply to this message or contact the journal office at ploscompbiol@plos.org. When you're ready to submit your revision, log on to https://www.editorialmanager.com/pcompbiol/ and select the 'Submissions Needing Revision' folder to locate your manuscript file.

Please include the following items when submitting your revised manuscript:

* A letter that responds to each point raised by the editor and reviewer(s). You should upload this letter as a separate file labeled 'Response to Reviewers'. This file does not need to include responses to formatting updates and technical items listed in the 'Journal Requirements' section below.

* A marked-up copy of your manuscript that highlights changes made to the original version. You should upload this as a separate file labeled 'Revised Manuscript with Track Changes'.

* An unmarked version of your revised paper without tracked changes. You should upload this as a separate file labeled 'Manuscript'.

If you would like to make changes to your financial disclosure, competing interests statement, or data availability statement, please make these updates within the submission form at the time of resubmission. Guidelines for resubmitting your figure files are available below the reviewer comments at the end of this letter

We look forward to receiving your revised manuscript.

Kind regards,

Lihua Zhang

Academic Editor

PLOS Computational Biology

Can Yang

Section Editor

PLOS Computational Biology

**Journal Requirements:**

If the reviewer comments include a recommendation to cite specific previously published works, please review and evaluate these publications to determine whether they are relevant and should be cited. There is no requirement to cite these works unless the editor has indicated otherwise.

1) Please ensure that the CRediT author contributions listed for every co-author are completed accurately and in full.

At this stage, the following Authors/Authors require contributions: Jia Song, and Qunhua Li. Please ensure that the full contributions of each author are acknowledged in the "Add/Edit/Remove Authors" section of our submission form.

The list of CRediT author contributions may be found here: https://journals.plos.org/ploscompbiol/s/authorship#loc-author-contributions

2) We ask that a manuscript source file is provided at Revision. Please upload your manuscript file as a .doc, .docx, .rtf or .tex. If you are providing a .tex file, please upload it under the item type u2018LaTeX Source Fileu2019 and leave your .pdf version as the item type u2018Manuscriptu2019.

3) Please upload all main figures as separate Figure files in .tif or .eps format. For more information about how to convert and format your figure files please see our guidelines:

https://journals.plos.org/ploscompbiol/s/figures

4) We have noticed that you have uploaded Supporting Information files, but you have not included a list of legends. Please add a full list of legends for your Supporting Information files after the references list.

5) Please amend your detailed Financial Disclosure statement. This is published with the article. It must therefore be completed in full sentences and contain the exact wording you wish to be published.

1) State the initials, alongside each funding source, of each author to receive each grant. For example: "This work was supported by the National Institutes of Health (####### to AM; ###### to CJ) and the National Science Foundation (###### to AM)."

2) State what role the funders took in the study. If the funders had no role in your study, please state: "The funders had no role in study design, data collection and analysis, decision to publish, or preparation of the manuscript."

3) If any authors received a salary from any of your funders, please state which authors and which funders..

If you did not receive any funding for this study, please simply state: u201cThe authors received no specific funding for this work.u201d

**Reviewers' comments:**

Reviewer's Responses to Questions

**Comments to the Authors:**

**Please note here if the review is uploaded as an attachment.**

Reviewer #1: In this paper, authors proposed a ST alignment method called OT-knn, wchich integrates local neighborhood information within an optimal transport framework. OT-knn was evaluated using simulated data with known ground-truth alignment and real datasets from multiple ST platforms. I have several major concerns:

1, The OT-kNN method is based on standard optimal transport (OT), which enforces full mass matching. Consequently, when applied to partially overlapping slices, it may lead to forced correspondences in non-overlapping regions. How is this issue handled in the experimental setup?

2，The cost matrix in OT only considers omics-based smooth features and does not incorporate spatial structural constraints. In contrast, PASTE introduces spatial relationships through the Gromov–Wasserstein (GW) distance, while SLAT preserves spatial topology via graph convolution.

In this case, would the current method potentially match spots that are highly similar in feature space but far apart in geometric space, thereby leading to spatially inconsistent or misaligned correspondences?

3，In OT-kNN, structural noise is simulated using a mild nonlinear transformation of the form (x^1.02,y^1.01). However, this transformation is separable along the two coordinate axes and does not introduce any local deformation, shear, or topological changes. Moreover, since the exponents are only slightly greater than 1, the transformation is numerically close to a linear mapping, resulting in a relatively small overall perturbation. Such a design is insufficient to capture the non-rigid deformations commonly observed in real biological tissues, such as local twisting, folding, or complex misalignment between slices. Under this limited nonlinear setting, the robustness of the method to structural noise may be overestimated. Furthermore, the experimental results presented in the manuscript do not provide convincing evidence that OT-kNN outperforms methods such as SLAT or DeST-OT under structural noise. In particular, experiments involving more complex structural inconsistencies are lacking.

4, The experiments presented in the manuscript only consider non-rigid transformations, and it is unclear whether the authors have evaluated the method under rigid transformations, such as rotation and translation between slices.

5, All experiments in the manuscript are conducted on datasets generated from the same platform. It remains unclear whether the proposed method can effectively align slices obtained from different sequencing platforms.

6, In the benchmarking experiments, the reproduction of SLAT obtains matching by applying optimal transport (OT) on the learned embeddings. However, this deviates from the original SLAT methodology, which determines correspondences directly based on embedding similarity. Why is OT applied as an additional step instead of using the original matching strategy proposed in SLAT? Notably, SLAT itself is designed to perform alignment through its embedding and matching mechanism, and introducing OT may alter its intended behavior and potentially affect the fairness of the comparison.

7, In the experiments on the DLPFC dataset, the results reported for PASTE are inconsistent with those presented in the original paper. This discrepancy raises concerns about the correctness of the implementation or the experimental setup used in this study.

8，The results in Fig. 3e show that increasing the number of neighboring nodes consistently improves model performance. However, in graph-based models, incorporating too many neighbors typically leads to over-smoothing, where node representations become indistinguishable and performance degrades. It is therefore unclear whether over-smoothing occurs in this setting, and how its impact on model performance is evaluated. The current results may not fully capture this effect.

9，It would be beneficial to include additional methods in the benchmarking experiments to provide a more comprehensive and convincing evaluation.

Reviewer #2: In this manuscript, the authors developed OT-knn, a neighborhood-aware optimal transport framework designed to align spatial transcriptomics (ST) data across different tissue slices. By reconstructing the gene expression profile of each spot based on its spatial k-nearest neighbors, the method aims to improve robustness to noise, spatial deformation, and biological heterogeneity. The authors systematically evaluated OT-knn on simulated and real datasets from 10x Genomics Visium, MERFISH, and Stereo-seq platforms, benchmarking it against methods such as PASTE, DeST-OT, and SLAT.

1. Methods such as PASTE can infer OT‑based pairwise correspondences across multiple slices to reconstruct 3D tissue. The authors should demonstrate whether OT‑knn’s neighborhood‑aware alignment leads to more accurate or more spatially consistent 3D stacking compared to existing approaches, or at least discuss its potential and limitations in this regard.

2. Beyond pairwise alignment, existing methods such as PASTE and SLAT have demonstrated the ability to integrate multiple slices for simultaneous spatial domain identification across slices. The authors should evaluate whether OT‑knn's multi‑slice alignment yields more consistent spatial domain, and evaluate it using ARI and NMI. Furthermore, the authors should benchmark OT‑knn against dedicated multi‑slice integration tools like STAligner (https://www.nature.com/articles/s43588-023-00528-w).

3. The simulation framework may not fully capture realistic spatial and transcriptional perturbations. The authors generate the second slice from a single human intestine slice using global spatial distortion and additive expression noise. Real tissue slices often exhibit local, non‑linear deformations and complex batch effects. I suggest the authors either validate on additional simulated scenarios (e.g., patch‑wise distortion, dropout noise) or explicitly discuss this limitation.

4. Optimal transport yields a probabilistic mapping between tissue slices, which is a core strength of OT-knn. However, the paper primarily visualizes the final discrete one-to-one matches (green/red plots). To help readers better understand the structure of the probabilistic transport plan, I suggest the authors add visualizations analogous to SLAT’s Fig. 3a and Fig. 4a for the real dataset.

5. Scalability of OT-knn to ultra-high-resolution technologies. Emerging platforms like Visium HD and Xenium generate data at subcellular or near-single-cell resolution, resulting in significantly more data points. The computational cost of building a kNN graph and solving an OT problem for hundreds of thousands of cells is nontrivial. The authors should discuss the expected runtime and memory scaling of OT-knn.

**********

**Have the authors made all data and (if applicable) computational code underlying the findings in their manuscript fully available?**

The PLOS Data policy requires authors to make all data and code underlying the findings described in their manuscript fully available without restriction, with rare exception (please refer to the Data Availability Statement in the manuscript PDF file). The data and code should be provided as part of the manuscript or its supporting information, or deposited to a public repository. For example, in addition to summary statistics, the data points behind means, medians and variance measures should be available. If there are restrictions on publicly sharing data or code —e.g. participant privacy or use of data from a third party—those must be specified.

Reviewer #1: Yes

Reviewer #2: Yes

**********

PLOS authors have the option to publish the peer review history of their article (what does this mean?). If published, this will include your full peer review and any attached files.

If you choose “no”, your identity will remain anonymous but your review may still be made public.

**Do you want your identity to be public for this peer review?**  For information about this choice, including consent withdrawal, please see our Privacy Policy.

Reviewer #1: No

Reviewer #2: No

**Figure resubmission:**

**Reproducibility:**

?>

# Author comment: Author response to Decision Letter 1

**DOI:** 10.1371/journal.pcbi.1014041.r002

27 Jul 2026

Attachment Submitted filename: Response to Reviewers.pdf

# Peer review: Decision Letter 1

**DOI:** 10.1371/journal.pcbi.1014041.r003

**Authors:** Can Yang, Lihua Zhang

1 Sep 2026

Dear Professor Li,

We are pleased to inform you that your manuscript 'OT-knn: a neighborhood-aware optimal transport framework for aligning spatial transcriptomics data' has been provisionally accepted for publication in PLOS Computational Biology.

Before your manuscript can be formally accepted you will need to complete some formatting changes, which you will receive in a follow up email. A member of our team will be in touch with a set of requests.

Please note that your manuscript will not be scheduled for publication until you have made the required changes, so a swift response is appreciated.

IMPORTANT: The editorial review process is now complete. PLOS will only permit corrections to spelling, formatting or significant scientific errors from this point onwards. Requests for major changes, or any which affect the scientific understanding of your work, will cause delays to the publication date of your manuscript.

Should you, your institution's press office or the journal office choose to press release your paper, you will automatically be opted out of early publication. We ask that you notify us now if you or your institution is planning to press release the article. All press must be co-ordinated with PLOS.

Thank you again for supporting Open Access publishing; we are looking forward to publishing your work in PLOS Computational Biology.

Best regards,

Lihua Zhang

Academic Editor

PLOS Computational Biology

Can Yang

Section Editor

PLOS Computational Biology

***********************************************************

Reviewer's Responses to Questions

**Comments to the Authors:**

**Please note here if the review is uploaded as an attachment.**

Reviewer #1: The authors have adequately addressed all of my concerns, and I have no further comments or questions.

Reviewer #2: The author have addressed my concerns, I don't have further comments.

**********

**Have the authors made all data and (if applicable) computational code underlying the findings in their manuscript fully available?**

The PLOS Data policy requires authors to make all data and code underlying the findings described in their manuscript fully available without restriction, with rare exception (please refer to the Data Availability Statement in the manuscript PDF file). The data and code should be provided as part of the manuscript or its supporting information, or deposited to a public repository. For example, in addition to summary statistics, the data points behind means, medians and variance measures should be available. If there are restrictions on publicly sharing data or code —e.g. participant privacy or use of data from a third party—those must be specified.

Reviewer #1: Yes

Reviewer #2: None

**********

PLOS authors have the option to publish the peer review history of their article (what does this mean?). If published, this will include your full peer review and any attached files.

If you choose “no”, your identity will remain anonymous but your review may still be made public.

**Do you want your identity to be public for this peer review?**  For information about this choice, including consent withdrawal, please see our Privacy Policy.

Reviewer #1: No

Reviewer #2: No

# Editor report: Acceptance letter

**DOI:** 10.1371/journal.pcbi.1014041.r004

**Authors:** Can Yang, Lihua Zhang

PCOMPBIOL-D-26-00380R1

OT-knn: a neighborhood-aware optimal transport framework for aligning spatial transcriptomics data

Dear Dr Li,

I am pleased to inform you that your manuscript has been formally accepted for publication in PLOS Computational Biology. Your manuscript is now with our production department and you will be notified of the publication date in due course.

The corresponding author will soon be receiving a typeset proof for review, to ensure errors have not been introduced during production. Please review the PDF proof of your manuscript carefully, as this is the last chance to correct any errors. Please note that major changes, or those which affect the scientific understanding of the work, will likely cause delays to the publication date of your manuscript.

Soon after your final files are uploaded, unless you have opted out, the early version of your manuscript will be published online. The date of the early version will be your article's publication date. The final article will be published to the same URL, and all versions of the paper will be accessible to readers.

For Research, Software, and Methods articles, you will receive an invoice from PLOS for your publication fee after your manuscript has reached the completed accept phase. If you receive an email requesting payment before acceptance or for any other service, this may be a phishing scheme. Learn how to identify phishing emails and protect your accounts at https://explore.plos.org/phishing.

Thank you again for supporting PLOS Computational Biology and open-access publishing. We are looking forward to publishing your work!

With kind regards,

Anitha Samidurai

PLOS Computational Biology | Carlyle House, Carlyle Road, Cambridge CB4 3DN | United Kingdom ploscompbiol@plos.org | Phone +44 (0) 1223-442824 | ploscompbiol.org | @PLOSCompBiol
